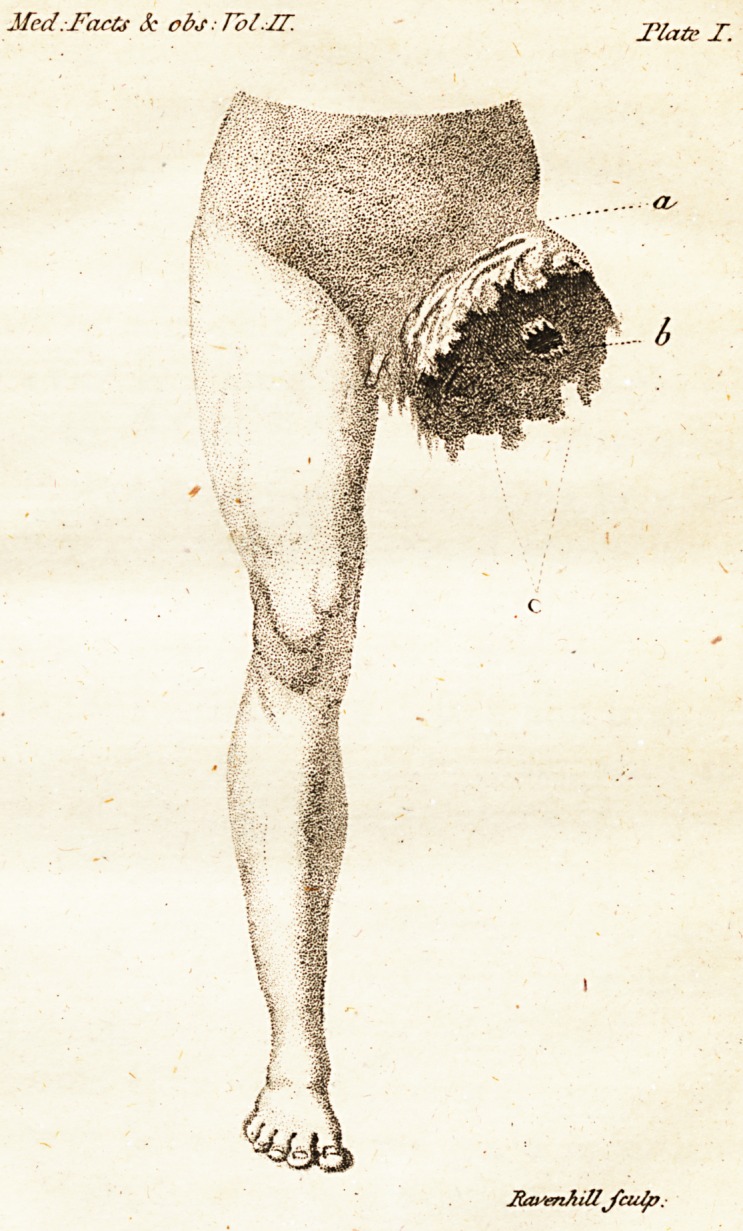# Case of a Boy Whose Left Leg and Thigh, Together with Part of the Scrotum, Were Torn off by a Slitting Mill

**Published:** 1792

**Authors:** Henry Yates Carter

**Affiliations:** Surgeon at Kettley, near Wellington, in Shropshire.


III. *Case of a Boy whose left Leg and Thigh, together with Part of the Scrotum, were torn off by a Slitting Mill. By the Same.*

ON the 24th of November, 1789, a boy, named Miles, aged about twelve years, in passing through the slitting mill at Kettley, unluckily put the great toe of his left foot between the pinion wheels while the mill was at work. The consequence was, that the toe fastened, and the limb was gradually drawn in, and crushed as it went, till the mill had reached as near to his body as where the sartorius muscle crosses one of the heads of the triceps. At that instant a man, hearing his cries, came to his relief, and forcibly tore him from the machine, by which means the muscular integuments were separated from his body, together with the whole of the left side of the scrotum. The other side also of the scrotum was lacerated, together with part of the pyramidalis muscle, the laceration extending from that ſide of the pubis in the direction of the line marked in the drawing*; the ſeparation of the external layer of muſcles on the poſterior ſide extending as far round as the gluteus medius.

When I attended, which was immediately after the accident, I found him laying on the floor covered with a blanket, and ſeemingly free from pain, or any anxiety † farther than what appeared to proceed from the trouble his parents were in in conſequence of the accident.

In almoſt any other caſe the firſt conſideration would have been to have ſearched for and ſecured the crural artery; but here a deviation from general practice, in caſes of amputation, became neceſſary, as a diligent ſearch would,

* See the reference at *a* in the annexed plate; *b* points out the part at which the bone was ſeparated; and *c* the lacerated remains of the faſcialis and glutæus maximus muſcles.

† An extraordinary inſtance to the contrary of this I ſaw in the memorable action on the 12th of April, 1782, on board the Ardent, (at that time a French ſhip) in a man who received a wound from a ſhot which took off the right thigh in a ſimilar manner to this; but who became delirious from the moment of the receipt of the injury, and expired in about twelve hours without any remiſſion of the delirium till within a very few minutes of his death.

Ravenhill sculp.

in all probability, have been attended with the utmoſt danger, inaſmuch as the whole of the remaining muſcular integuments, as far up as where the bone was divided, were violently lacerated, and the whole cruſhed together in a maſs. For this reaſon, and as there was no hæmorrhage preſent, I did not think it prudent to remove any part of the adhering muſcles with a view of finding the artery, as the chances in ſuch a caſe ſeemed to be conſiderably againſt the probability of diſcovering it but by a fatal hæmorrhage: and it appeared reaſonable to ſuppoſe that a portion of the artery had been drawn out and carried away with the general maſs in the exertion uſed to free him from the machine; and that this, together with the violence of the preſſure upon ſuch a body in ſo ſmall a ſpace, and the contraction uſual in ſuch caſes, had perhaps (may I ſay undoubtedly had?) cloſed the mouths of the veſſels in the muſcular integuments that remained. The artery, even if it could have been diſcovered, would perhaps have been ſo near the groin as to have rendered the ſecuring it a work of much difficulty, if not totally impracticable, and therefore certainly fatal. For theſe reaſons, and finding (notwithſtanding he had been much

C 2 diſturbed difturbed by his removal home) no appearance of hæmorrhage, I thought myfelf highly juf-tifiable in the omiffion.

I moreover determined in my own mind not to attempt even a feparation of any of the lacerated parts, except fuch as from their fituation fhould be more immediately in the way of dreffing, and from the removal of which there could be no danger.

He was laid on a mattrefs, and bolftered as much upon his right fide as could be admitted with any degree of eafe; that part of his body was alfo raifed confiderably higher than the other. Before the lacerated ftump was wholly placed together I thought it prudent to take as much care, as the nature of the cafe would admit, to prevent a future hæmorrhage, by endeavouring by fome kind of ftricture to leffen the effects of the blood's velocity on the part. For this purpofe a broad roller or bandage was paffed round his body, and properly fecured; and to this another broad piece was faftened on the back part near the right os innominatum. The integuments were then placed together, round the end of the bone, and, after being well covered with lint dipped in a mixture of equal parts of camphorated fpirit of wine and

ſpirit of turpentine, were ſprinkled well with wheat flour, over which another layer of lint pledgits and a ſuitable compreſs were applied. A bolſter was then placed upon that part where the inguinal artery paſſes out of the groin, and the bandage being brought under the right thigh, and carried over the bolſter, was faſtened upon the other hip. The preſſure which this occaſioned ſeemed to be as conſiderable as could with prudence be admitted.

The boy ſeemed to have ſuffered very little fatigue from the dreſſing, and was in good ſpirits. He was permitted to have a ſmall quantity of weak wine and water for the preſent, and was ordered barley water acidulated with lemon juice, and balm tea, for his common drink. In the evening an opiate was adminiſtered, in order, as much as poſſible, to guard againſt ſpaſm, of which there appeared reaſon to be apprehenſive.

On the 25th I viſited him early in the morning, and, contrary to my expectation, found him not only alive, but in excellent ſpirits. There was no diſcharge of blood from the dreſſings; there was a gentle warmth on the parts: he had reſted conſiderably, was free from ſevere pain, and had very little fever.

The whole of theſe circumſtances, ſo unexpectedly favourable, gave me ſome hopes, if a diſcharge could be procured, a kindly ſeparation of the ſloughs produced, the fever moderated, and his ſtrength ſupported, that there might be a chance of his recovery.

A ſtool was procured by means of a clyſter; and he was directed to take occaſionally of a ſaline mixture, to which were added ſmall doſes of opium. His diet conſiſted of ſago, boiled rice, chicken broth, and ſalep.

On the 26th I found that he had reſted well; was perfectly ſenſible; free from pain; had taken conſiderable nouriſhment, and appeared extremely compoſed. His fever, notwithſtanding, was a little increaſed. He was now directed to take liberally of a decoction of bark, and an opiate was adminiſtered in the evening.

On the morning of the 27th I found him complaining of a ſoreneſs of the parts; he was ſenſible; had reſted conſiderably; but did not appear ſo perfectly compoſed as hitherto; his fever, however, was much abated, and upon turning down the dreſſings I found the appearance of matter beginning to form.

On the 28th the patient was apparently weaker. He had paſſed a reſtleſs night; complained much of thirſt and head-ach ; and was frequently ſeized with ſlight ſhivering. He had had one ſtool ſince yeſterday, and now took red bark in ſubſtance, added to the decoction, with elixir of vitriol ; and drank port wine.

On the 29th I found that he had had a reſtleſs night, and had been at times delirious. In the morning, however, he took nouriſhment, and ſeemed eaſy and compoſed, his pulſe indicating no very great degree of irritability : he ſpoke at the ſame time ſenſibly, though faintly ; but died that day about noon.

This caſe, though it may not be capable of much uſeful application, ſeems to be highly deſerving of being recorded as a curious and extraordinary fact. I can find only two inſtances of an accident at all analogous to it in books. Of each of thoſe the event was ſucceſsful ; but as in both it was the arm inſtead of the thigh that was torn off, it may, perhaps, be reaſonable to preſume that a greater degre of danger attends ſuch an accident in the lower than in the upper extremities of the body. One of the two caſes is that deſcribed by Mr. Cheſelden in his Anatomy of the Human

Body *, (where he has given a figure of the patient), and by Mr. Belchier in the Philosophical Tranfactions †, of Samuel Wood, a Miller, whofe arm, with the fcapula, was torn off from his body by a rope winding round it, the other end being faftened to the coggs of a windmill. This happened in the year 1737.

The other cafe occurred, in 1776, to Mr. James Carmichael, Surgeon at Port Glafgow, and is inferted in the fifth volume of Dr. Duncan's Medical Commentaries. The fubject of it was a girl, three years and a half old, who was entangled, with an apron pinned about her fhoulders, by the fpindle of a barley mill, going at its full career ‡, and twifted round it with equal velocity. In this cafe the left arm was torn off an inch and a half above the elbow, and the mufcles and integuments were much lacerated higher up.

It is worthy of remark, that in neither of

* Sixth Edition, 8vo. London, 1741. page 321.

† Vol. XL. page 313.

‡ From an accurate calculation, Mr. Carmichael found that the fpindle of the mill flew round 140 times in a minute, when working with its full force.

theſe two caſes, any more than in the one I have related, was there a diſcharge of blood. In the Miller a cure was completed without the leaſt appearance of hæmorrhage, and without a ſingle blood veſſel having been taken up; and Mr. Carmichael relates of his patient, that when he firſt ſaw her, an hour after the accident, he was aſtoniſhed to find that ſhe had not loſt a ſpoonful of blood. How long the abſence of hæmorrhage would have continued in this caſe could not be determined, as the injury done to the parts above the elbow rendered the amputation of the arm at the ſhoulder neceſſary, as affording, ſeemingly, the only probable chance of ſaving the life of the patient. The operation was accordingly performed, and the cure completed in little more than two months.

## Figures and Tables

**Figure f1:**